# Laryngeal Dimensions: A Computed Tomography Study

**DOI:** 10.1055/s-0045-1810117

**Published:** 2025-10-09

**Authors:** Mohammad Waheed El-Anwar, Ali Awad, Atef Hussain, Ehsan Hendawy, Mohammad A. El Shawadfy, Mohamed Ibrahim Abdelzeem Heggy, Mohamed Adel Mobasher

**Affiliations:** 1 Department of Otolaryngology–Head and Neck Surgery, Faculty of Medicine, Zagazig University, Zagazig, Egypt; 2 Department of Otorhinolaryngology, Faculty of Medicine, Al Aazhar University, Cairo, Egypt

**Keywords:** vocal fold, larynx, subglottic, thyroid cartilage, computed tomography

## Abstract

**Introduction:**

The computed tomography (CT) details of the dimensions of the larynx are not fully covered in the literature, so it is important to build up a database for the CT measurements of that area in different ethnic groups. Preoperative details provided by CT are crucial prior to any procedure or approach involving the larynx.

**Objective:**

To measure and report the different dimensions of the larynx and vocal cords through CT.

**Methods:**

We analyzed 100 CTs of the larynx (200 vocal cords): axial images were acquired through multiplanar reformation to obtain minute details of all subjects.

**Results:**

The mean anteroposterior (AP) vocal fold length (VFL) was of 16.8 ± 2.6 (range: 11.8–22.9) mm, which was significantly longer in male subjects (mean = 18.4 ± 2.2 mm; range = 10.9–19.8 mm) compared to female subjects (mean = 15.3 ± 1.95 mm; range = 10.9–22.9 mm). The mean width of the subglottic area was of 14.88 ± 2.18 (range = 8.9–21.1) mm, which was significantly wider in male subjects (15.6 ± 2.13 mm; range = 9.1–21.1 mm) compared to female subjects (mean = 14.18 ± 1.99 mm; range = 8.9–17.8 mm). There was a positive correlation between the AP and transverse diameters of the subglotic area with the VFL: the longer the AP and transverse diameters of the subglottic area, the longer the VFL and vice versa.

**Conclusion:**

The present study improves the awareness of surgeons and radiologists of the laryngeal dimensions, and it can be of help to residents in training.

## Introduction

The methods developed for the management of laryngeal disorders in laryngology and phoniatrics have led to an increasing demand for more individualized, real, and accurate measurements of glottic structures. Measurements of the glottic area and its structures provide precious data for the assessment of the various laryngeal pathologies and of some phonosurgeries and procedures, such as correction of glottic insufficiency following laryngeal palsy.

Endoscopic laryngeal imaging does not enable adequate absolute measurements of the glottic area or length because both flexible and rigid laryngeal endoscopes have a fairly broad field depth, resulting in the expectation of large alterations in the distance from the endoscope to the glottic area through examination with varying measurements. There is also some distortion of the laryngeal images depending on the angle between the instruments and the glottic area and of the endoscopy system itself. Several authors have discussed these sources of errors and proposed standardized examination procedures to lessen the errors and carry out reproducible examinations. 1 2 3 Moreover, accurate absolute measurements from laryngeal examination video recording demand a known calibration distance to relate.

Computed tomography (CT) is a standard tool for the assessment of laryngeal neoplasms and trauma. 4 5 Axial scans obtained during phonation at the plane of the true vocal fold (VF) can identify soft tissue and cartilage. The length of the membranous part of the VF (from the anterior commissure to the tip of the vocal process) is invariable if the subjects phonate at a stable, constant pitch in a stable voice recorder. 6 Thus, CT measurement of the VF length (VFL) might be used to calibrate and estimates of VFL from stroboscope video recording and so the glottic region during phonation can also be estimated.

Therefore, preoperative CT assessment is essential to identify the anatomical landmarks and dimensions of the larynx, their relationships to the surrounding structures, and to evaluate the exact extension of the disease to aid in the proper management and the performance of a safe and effective surgery. But, in certain cases, such as those of neoplasms, trauma, or revision, these landmarks may be absent or distorted, increasing the dissection difficulty and the risk of complications. Therefore, it is paramount to identify the dimensions of the larynx preoperatively and its landmarks to guide the surgeons.

In the literature, some attempts to measure the VFL have been made in cadavers, 7 in a recently removed larynx, 8 or based on X-rays. 9 10 The results obtained from cadavers could not reflect the physiological VFL in a live person, so they do not provide helpful clinical information. Although using lateral X-rays of the neck to estimate the VFL might be convenient and provide practical data, it is difficult to measure the VFL precisely, and vague landmarks are used to estimate the VFL. In lateral radiographs, the VF thickness renders the reference points obscure and makes data interpretation hard.

Thus, the present study aimed to provide more precise measurements of the VFL and of the subglottic area in live subjects through CT. The results may influence laryngeal surgery, especially the endoscopic approach and phonosurgery.

## Methods

The current retrospective study was conducted on 100 CTs of the larynx (200 vocal cords) at the Otorhinolaryngology Departments of two universities between July 2021 and January 2023. The informed consent form was signed by all subjects, and the study was approved by the institutional review board.

Patients younger than 18 years, with a history of trauma or surgery in the larynx or neck, and those with congenital anomalies and/or malignancies were excluded.

All subjects were submitted to 64-slice CT scans (Lightspeed VCT 64, GE HealthCare). The protocol of the 64-slice multidetector CT (MDCT) scans were as follows: 0.625 mm of detector width, sections with a width of 1.5mm, and a 0.5-mm interval reconstruction. 130KV and 150 mA/sec with 1.5sec scan time.

The axial scans of the larynx were performed with the subject in the supine position and breathing quietly. A high-resolution algorithm was used to enhance the appearance of the delicate bony details.

**Fig. 1:**
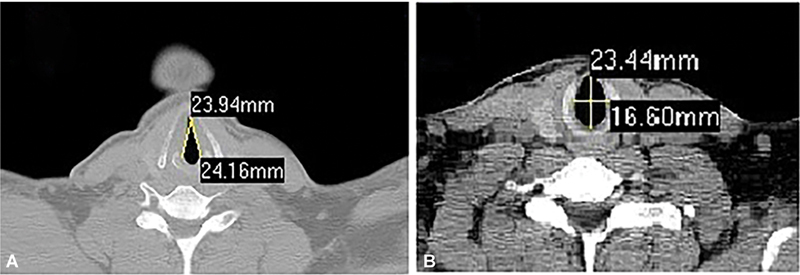
Measurements of the length of the vocal folds ( **A** ) and of the dimensions of the subglottic area ( **B** ).

The scans were reviewed in a routine standardized manner in order not to miss small details. The VFL from the anterior commissure to the tip of the vocal process was measured. The anteroposterior (AP) and transverse dimensions, along the axial planes, were measured at the level of the cricoid cartilage ( Fig. 1 ). The statistical analysis was conducted using IBM SPSS Statistics for Windows (IBM Corp.) software, version 25.0. Values of *p* < 0.05 were considered statistically significant.

## Results

The present study included 100 CT scans (200 sides) of 48 male (48%) and 52 female subjects (52%) with a mean age of 53.4 (range:13–87) years.

The mean AP VFL was of 16.8 ± 2.6 (range: 12.5 to 22.9) mm ( Table 1 ), which was significantly longer in male (mean = 18.4 ± 2.2 mm; range = 15.6–12.9 mm) compared to female subjects (mean = 15.3 ± 1.95 mm; range = 12.5–19 mm) (t = 7.4687; *p* < 0.0001) ( Table 1 ).

**Table 1:** Computed tomography measurements of the length of the vocal cords and dimensions of the subglottic area

	Mean vocal cord m length (mm)	Mean subglottic area dimensions	
		Transverse (mm)	Anteroposterior (mm)
**All subjects**	16.8 ± 2.6	14.88 ± 2.18	17.5 ± 2.9
**Males (n = 48)**	18.4 ± 2.2	16.4 ± 2.46	18.7 ± 2.9
**Females (n = 52)**	15.3 ± 1.95	14.3 ± 1.8	16.49 ± 2.47
**Student's** ***t*** **-test**	7.4687	3.4466	4.1124
***p*** **-value**	< 0.0001	0.0008	< 0.0001

The mean AP diameter of the subglottic area was of 17.5 ± 2.9 (range = 12.7–24.9) mm, which was longer in male (mean = 18.7 ± 2.9 mm; range = 14.2–24.9 mm) compared to female subjects (mean = 16.49 ± 2.47 mm; range = 12.7–20.8 mm)

The mean width at the level of subglottic area was of 14.88 ± 2.18 (range: 12.3–21.1) mm, and it was significantly wider in male (mean = 16.4 ± 2.46 mm; range: 13.2–21.1 mm) compared to female subjects (mean = 14.3 ± 1.8 mm; range: 12.3–17.8 mm) (t = 4.8979; *p* < 0.0001) ( Table 1 ). Therefore, all the dimensions of the larynx were found to be significantly smaller in female compared to male subjects.

There was strong positive correlation between the AP diameter of the subglottic area and the VFL: the longer the AP diameter of the subglottic area, the longer the VFL and vice versa. (r = 0.7181; *p* < 0.0001)

There was moderate positive correlation between transverse diameter of the subglottic area and the VFL: the longer the transverse diameter of the subglottic area, the longer the VFL and vice versa (r = 0.6718; *p* < 0.0001).

## Discussion

The VFL can be used as calibration distance to assess the absolute area and length of glottal structures and/or masses or lesions based on an examination with flexible or rigid laryngeal endoscopes and on video recordings during phonation.

The dimension of the membranous glottis appears to be an appropriate calibration length because its anatomical landmarks could be accurately determined by the laryngoscopic examination and based on the CT scans.

Laryngoscopy is considered the standard tool for laryngeal examination; it is a simple and valued option for the assessment of laryngeal lesions or the glottic gap, but it is not suitable for the measurement of the VFL or of the dimensions of lesions. This limits its quality for the measurement of the absolute areas.

The length of the outstretched VF can also be observed and measured distinctly through direct laryngoscopy under general anesthesia. But, under general anesthesia, there are dimension differences that could arise from the muscle tone loss or stretch by the endotracheal tube insertion or possibly caused by the introduction of the laryngoscope into the larynx. All these aspects could change the VF shape and could cause VF thinning and widening of the base of a benign lesion. 11 This factor should be taken into consideration when performing functional VF surgeries under general anesthesia. That is why the CT measurement appears to be more real, reliable, and accurate.

Knowledge of the VFL is significant for professionals who rely on their voices for work, such as teachers and singers. However, a simple and risk-free method that enables the collection of normative reference values for the laryngeal structures has not yet been fully developed. 12

Improved optic instruments, such as flexible or rigid laryngoscopes with video recording provide a superior view of the anatomy of the surface of the larynx. If stroboscopy is used, the glottic vibrations at the free VF edges are distinctly seen during phonation, and the degree of glottic closure can be estimated. Moreover, the disturbance of the VF vibrations can be instantly examined during phonations. 13 14 Videostroboscopy is more sensitive to camera rotation, the side movement of the laryngoscope, and patient movements, which give the VF delocation. 15

However, the endoscopic laryngeal images obtained with these tools do not provide an adequate absolute measurement of glottic area or length because of the fairly wide depth of field for flexible and rigid laryngeal endoscopes, with large distance variations between the endoscope and the VF throughout the examination, causing varied area and length measurments. In addition, there are significant distortions in the larynx images depending on the angle between the endoscope and the VF and on the endoscope, system used (called *barrel-shaped distortions* ). 2 13

Various methods have been used in the past to measure VFL 7 in cadaveric larynges, but they often used a fixation or plastination process for the specimens. Hu et al. 9 have attempted to measure the true VFL in live individuals using methods such as photography, plain films, ultrasound, and laser. Kim et al. 10 used the X ray but could assess only the laryngeal airway, not the inner structure of the larynx. Litman et al. 16 used magnetic resonance imaging (MRI) scans to measure the larynges of children under sedation, which could alter the dimensions. Moreover, the unavailability of the MRI and patient fears regarding the device make it difficult to depend on it. And Sankar et al. 17 used a three-dimensional digital model of the human larynx derived from the published literature and radiographs.

Computed tomography (CT) has become one of the gold-standard tools for laryngeal evaluation, providing high-quality axial scans of true VF and of the laryngeal cartilage. Laryngeal CT is a standard method to evaluate neoplastic and traumatic laryngeal lesions. The axial scans obtained during phonation at the plane of the true VF could clearly identify laryngeal soft tissue and cartilage. The length of the membranous part of the VF (from the tip of the vocal process to the anterior commissure) is invariable when the subject phonates at a stable, constant pitch and in a stable voice register. 10

Thus, CT measurements of the real VFL, from the vocal process to the anterior commissure, could be used to estimate the VFL through stroboscopic video recordings in which the laryngeal structures and glottal area can be identified and estimated. 18

The advantages of measuring the absolute VFL through CT are that the measurements are easy and accurate, with the subject breathing quietly and without the introduction of instruments in the air passage that may disturb it or cause patient discomfort, and without need for local anesthetics or any procedures through the airways. A disadvantage is the radiation dose, which is minimal, because high-resolution CT scans of the neck region are associated with a relatively small dose of radiation, which makes this type of scanning technique safe. 5 19

Therefore, we chose CT to determine the different dimensions of the larynx and VF. In the current study, the mean AP VFL was of 16.8 ± 2.6 (range: 11.8–22.9) mm, which was significantly longer in male subjects (mean = 18.4 ± 2.2 mm; range = 10.9–19.8 mm) compared to female subjects (mean = 15.3 ± 1.95 mm; range = 10.9–22.9 mm). The mean width of the subglottic area was of 14.88 ± 2.18 (range = 8.9–21.1) mm, which was significantly wider in male subjects (15.6 ± 2.13 mm; range = 9.1–21.1 mm) compared to female subjects (mean = 14.18 ± 1.99 mm; range = 8.9–17.8 mm). There was a positive correlation between the AP and transverse diameters of the subglotic area with the VFL: the longer the AP and transverse diameters of the subglottic area, the longer the VFL and vice versa.

Previous studies have measured the mean VFL in healthy male and female adults, and the respective results were as follows: 13.8 ± 2.92 mm and 10.7 ± 1.63; 20 22.09 ± 3.07 mm and 17.55 ± 0.92 mm; 21 15.3 mm and 13.5 mm; 9 16.11 ± 2.62 mm and 14.10 ± 1.54 mm; 22 and 24.9 mm and 17.5 mm. 8 In all of these studies, it was clear that male subjects has longer VFL than female subjects, but there are wide variations in the measured VFL in different studies and regions, reflecting the importance of normative data for each ethnic group. The mean VFL is of 14.9, 16.0, 16.6, 18.4, 19.5, and 20.9 mm for sopranos, mezzo-sopranos, altos, tenors, baritones, and basses, respectively. 23 These variations reflect the importance of individual VFL measurements for each subject.

Hertegard et al. 18 repeated CT scans for several pitches in one of their male subjects, who showed a maximum variation in the measured length of the membranous part of the glottis of 0.35 mm, that is 3% more. This male subject and a female subject were examined through CT during phonation with normal and loud intensity in the frequencies 110 Hz and 220 Hz respectively. For the male subject, they found no length difference, but the female subject presented a 0.5-mm shortening of the membranous part of the glottis during loud phonation compared to normal phonation. Therefore, the differences in FVL during phonation are minimal and insignificant.

In addition, measurements of the dimensions of subglottic area at the cricoid cartilage is are highly valuable and important because it is the only complete ring in the airway representing the maximum caliber through which the endoscopy or endotracheal tube can be passed, and can be considered as the main cartilage around which the larynx framework is made-up. The dimensions measured in the current study could be highly valuable for the evaluation of the vocal cord range and also for the planning of laryngeal surgeries.

## Conclusion

The present study updates the knowledge of laryngeal dimensions from a CT perspective to improve surgeons' and radiologists' awareness for optimum and safe surgeries.
